# Opposite latitudinal patterns for bird and arthropod predation revealed in experiments with differently colored artificial prey

**DOI:** 10.1002/ece3.5862

**Published:** 2019-11-25

**Authors:** Elena L. Zvereva, Bastien Castagneyrol, Tatiana Cornelissen, Anders Forsman, Juan Antonio Hernández‐Agüero, Tero Klemola, Lucas Paolucci, Vicente Polo, Norma Salinas, Kasselman Jurie Theron, Guorui Xu, Vitali Zverev, Mikhail V. Kozlov

**Affiliations:** ^1^ Department of Biology University of Turku Turku Finland; ^2^ BIOGECO INRA Univ. Bordeaux Cestas Cedex France; ^3^ Departamento de Genética, Ecologia e Evolução Universidade Federal de Minas Gerais Belo Horizonte Brazil; ^4^ Department of Biology and Environmental Science Linnaeus University Kalmar Sweden; ^5^ Department of Biology and Geology, Physics and Inorganic Chemistry University Rey Juan Carlos Móstoles Spain; ^6^ Setor de Ecologia e Conservação Departamento de Biologia Universidade Federal de Lavras Lavras Brazil; ^7^ Instituto de Pesquisa Ambiental da Amazônia Brasília Brazil; ^8^ Departamento de Biologia Geral Universidade Federal de Viçosa, Campus Universitário Viçosa Brazil; ^9^ Instituto de Ciencias de la Naturaleza, Territorio y Energías Renovables Pontificia Universidad Católica del Perú Lima Peru; ^10^ Department of Conservation Ecology and Entomology Stellenbosch University Matieland South Africa; ^11^ CAS Key Laboratory of Tropical Forest Ecology Xishuangbanna Tropical Botanical Garden Chinese Academy of Sciences Menglun China

**Keywords:** arthropod predators, artificial prey, avian predators, biotic interactions, color preference, latitudinal pattern, plasticine models, predation rate

## Abstract

The strength of biotic interactions is generally thought to increase toward the equator, but support for this hypothesis is contradictory. We explored whether predator attacks on artificial prey of eight different colors vary among climates and whether this variation affects the detection of latitudinal patterns in predation. Bird attack rates negatively correlated with model luminance in cold and temperate environments, but not in tropical environments. Bird predation on black and on white (extremes in luminance) models demonstrated different latitudinal patterns, presumably due to differences in prey conspicuousness between habitats with different light regimes. When attacks on models of all colors were combined, arthropod predation decreased, whereas bird predation increased with increasing latitude. We conclude that selection for prey coloration may vary geographically and according to predator identity, and that the importance of different predators may show contrasting patterns, thus weakening the overall latitudinal trend in top‐down control of herbivorous insects.

## Graphical abstract

We explored whether predator attacks on artificial prey of eight different colors vary among climates and whether this variation affects the detection of latitudinal patterns in predation. We found that selection for prey coloration varies geographically and according to predator identity. When attacks on models of all colors were combined, arthropod predation decreased, whereas bird predation increased with increasing latitude.

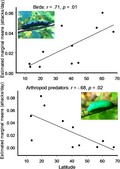

## 1 INTRODUCTION

Global patterns in the intensity of trophic interactions have recently become a subject of heated debate (Moles & Ollerton, 2016). Plant–herbivore interactions have received considerable attention (e.g., Kozlov, Lanta, Zverev, & Zvereva, 2015; Moles, Bonser, Poore, Wallis, & Foley, 2011; Moreira, Abdala‐Roberts, Parra‐Tabla, & Mooney, 2015), whereas predator–prey interactions remain less explored (Björkman, Berggren, & Bylund, 2011). Although the prevailing view seems to be that biotic interactions become more intense at lower latitudes (Adams & Zhang, 2009; Pennings & Silliman, 2005; Roslin et al., 2017; Schemske, Mittelbach, Cornell, Sobel, & Roy, 2009), many studies of both plant–herbivore (reviewed in Moles & Ollerton, 2016) and predator–prey (Lövei & Ferrante, 2017) interactions have reported no clear latitudinal pattern in the strength of these interactions. The inconsistent outcomes of these studies may partly result from the variety of methods used to estimate intensity of trophic interactions (Anstett, Nunes, Baskett, & Kotanen, 2016; Roslin et al., 2017).

To overcome the potentially distorting effects of nonuniform methods, Roslin et al. (2017) conducted a global study on geographical patterns in predator–prey interactions, based upon exposure of identical plasticine caterpillars at multiple study sites. In line with theoretical predictions (Schemske et al., 2009), the observed predation rates decreased from low to high latitudes. However, this decrease was due to changes in arthropod predation, whereas bird predation showed no statistically significant latitudinal trend (Roslin et al., 2017). The latter result seems surprising, because birds in tropical forests were estimated to consume 2.5 times more arthropod biomass (per hectare per year) when compared with birds in temperate and boreal forests, and 25 times more biomass when compared with birds in arctic tundra (Nyffeler, Sekercioglu, & Whelan, 2018). Therefore, the predation pressure that birds impose on herbivorous insects could be expected to reach its maximum in the tropics. This disagreement regarding the global pattern in bird predation, as obtained by different methods, calls for a deeper exploration of methodological sources of variability in estimates of bird predation.

In addition to the methods employed, the biological and ecological sources of variation may have contributed to the different outcomes in studies that explored latitudinal patterns in predator–prey interactions. Attack rates on model prey with the same appearance may vary among predator individuals and species, owing to differences in perception, experience, preference, and hunger level, as well as the ability of predators to recognize and discriminate prey. Predation rates also depend on many environmental characteristics, such as visual backgrounds and light conditions, which differ considerably among habitats (Endler, 1993; Ruxton, Allen, Sherratt, & Speed, 2018). In particular, prey visual appearance is subject to substantial temporal and spatial changes due to background and ambient light availability (Théry & Gomez, 2010). Therefore, variation in the illumination regime, both between and within habitats, may cause considerable differences in predation rates on the same kind of prey. For example, predation intensity on the same object can differ between illuminated and shady environments (Cheng et al., 2018; Rojas, Rautiala, & Mappes, 2014), and variations in ultraviolet light may considerably affect the search behavior of insectivorous birds (Church, Bennett, Cuthill, & Partridge, 1998).

Bird responses to prey that differ in appearance, including coloration, are influenced by previous experience (Ruxton et al., 2018). The experience of birds in a certain habitat depends on the composition of the local prey community, and particularly on the variability in coloration of local prey species, which may differ between tropical and temperate sites (Adams, Kang, & June‐Wells, 2014). Great environmental variability was found to reduce the avoidance of novel foods by birds (Greenberg & Mettke‐Hofman, 2001, and references therein). These results suggest that birds in tropics—the region with the highest biodiversity (Willig, Kaufman, & Stevens, 2003)—will accept a wider range of prey types, and a wider range of colors in particular, when compared with birds in other biomes.

Rates of predation on differently colored prey in natural environments may also depend on the time relative to bird's breeding season, because young naïve birds strongly differ from adult birds in their responses to a certain prey appearance (Ruxton et al., 2018; Mappes, Kokko, Ojala, & Lindström, 2014). In addition, the responses of predators to prey coloration depend on the characteristics of predator color vision, which differ considerably both between major groups of predators (arthropods, birds, mammals, and reptiles) and within these groups (Théry & Gomez, 2010). Among animals preying on insects, discrimination of colors plays major role in predatory behavior of birds (Théry & Gomez, 2010). Arthropod predators rely mostly on chemical cues in prey search and discrimination (Traniello, 1989; Zvereva & Kozlov, 2016); nevertheless, many arthropod species possess color vision (Briscoe & Chittka, 2001), and some of them use prey coloration in their foraging behavior (Taylor, Maier, Byrne, Amin, & Morehouse, 2014). We therefore suggest that the use of a set of different prey items (instead of prey of one type) would generate more robust inferences regarding variation in predation rates among different environments.

In this study, we endeavored to advance the understanding of factors shaping global latitudinal pattern in top‐down control of herbivorous insects. We conducted experiments with model prey of eight different colors to test the following hypotheses: (a) Predators differentially attack prey of different colors; (b) avian and arthropod predators differ in their responses to colors of model prey; (c) the attack rates on model prey of different colors vary among sites according to the latitude and climate of the site; and (d) the overall rates of predation decrease from low to high latitudes.

## 2 MATERIALS AND METHODS

### 2.1 Experimental design

**Figure 1:**
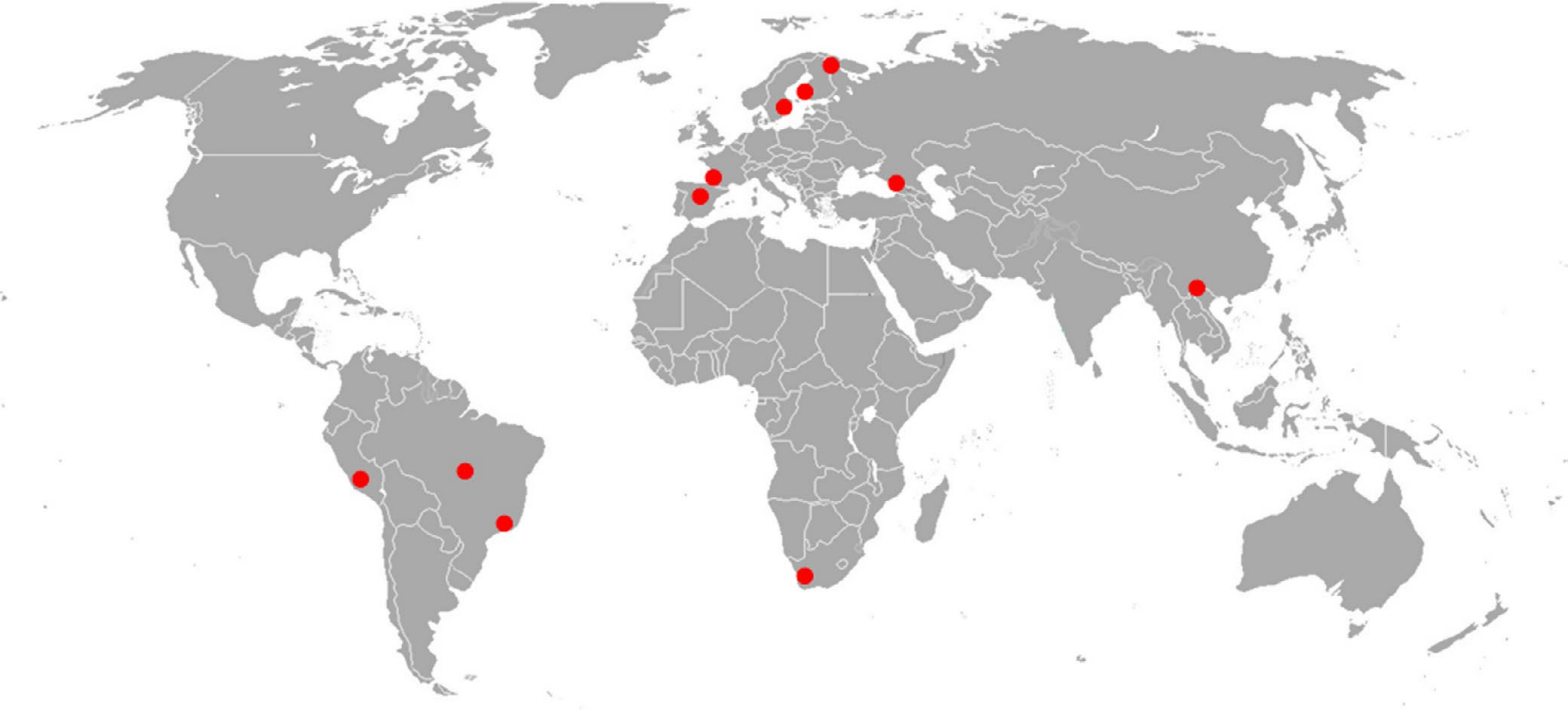
Location of the study sites. For more information, consult Table A1 in Appendix

The experiments were conducted in 2017–2018 using a standardized method in 11 sites worldwide, from 33°57′S to 67°38′N (Figure 1), representing cold (three sites), temperate (five sites), and tropical (three sites) climates; climatic zones were distinguished based on average midsummer temperatures (Table A1 in Appendix). The study sites were selected in natural forest environments representative for each geographic zone; more details of vegetation type in each site are provided in Table A1. At each site, five mature individuals of each of the three most common woody species (15 trees in total) were haphazardly chosen for the experiment; the selected trees were situated more than 5 m apart.

**Figure 2:**
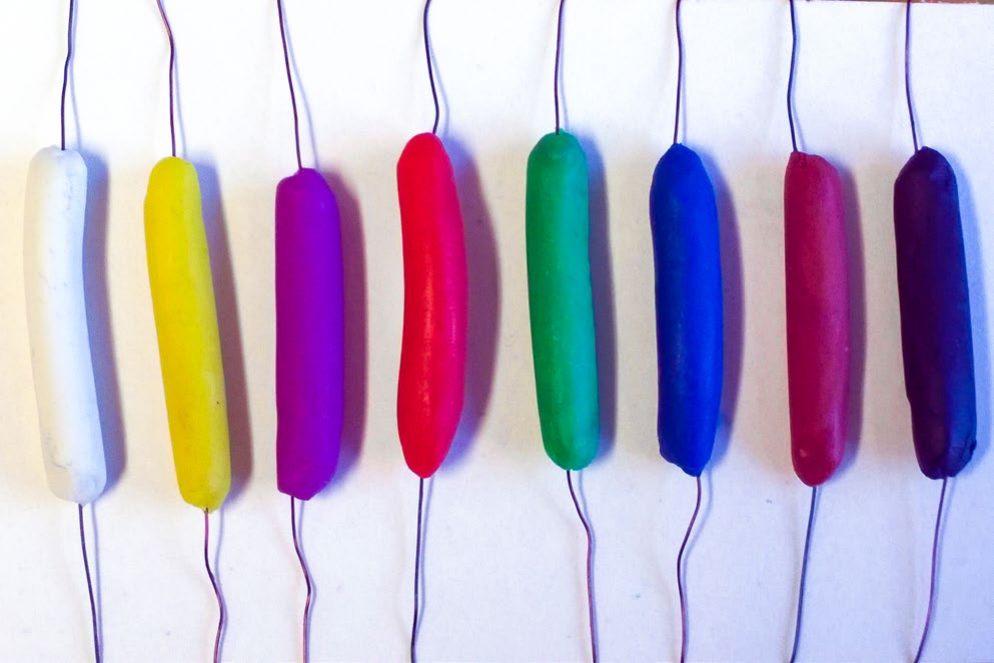
A set of plasticine caterpillars of eight different colors. This set was established on each of 15 trees per study site

**Figure 3:**
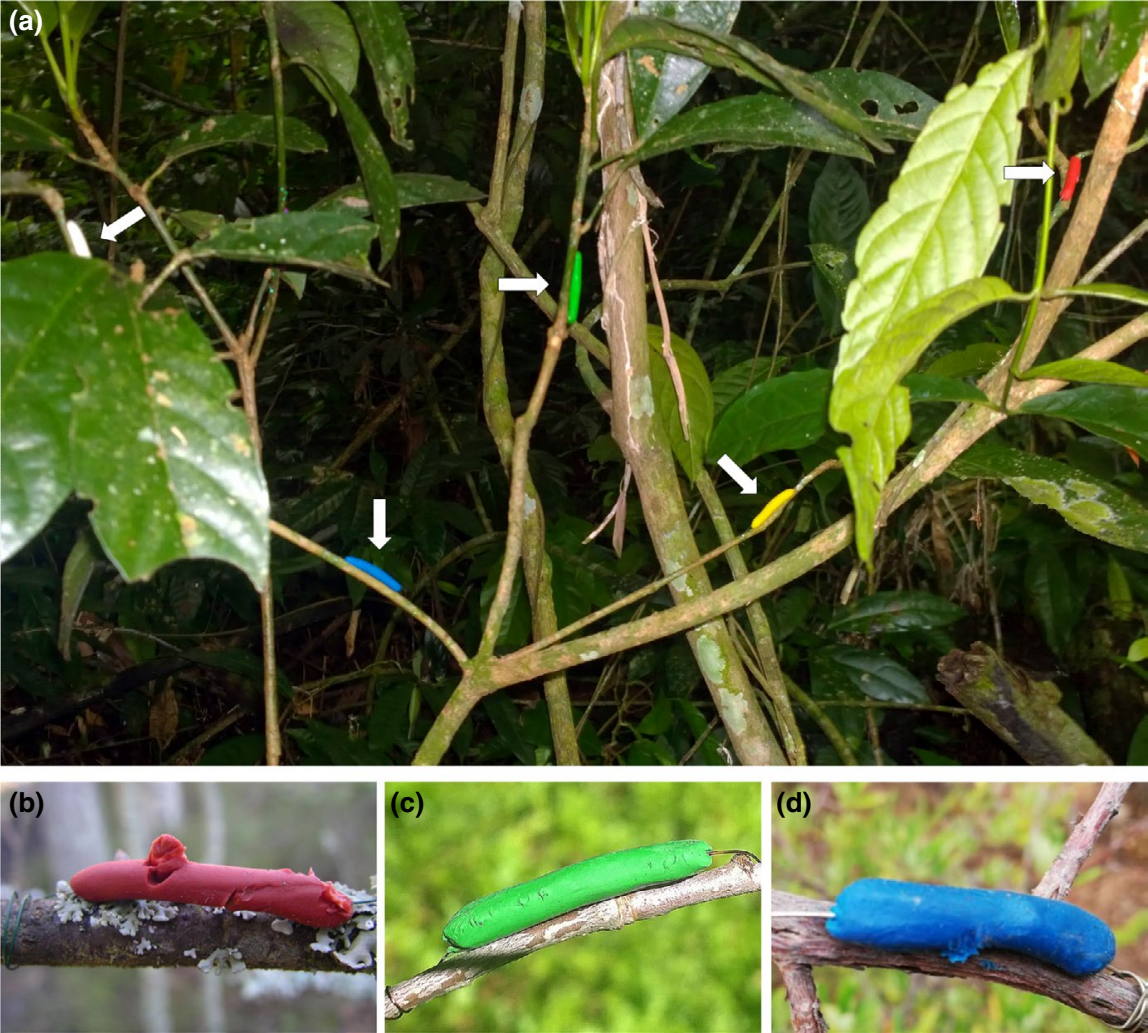
Examples (a) of the location of model caterpillars within a tree, five of eight colors are visible (site in Xishuangbanna, China) and of predation marks left by (b) birds, (c) arthropods, and (d) mammals

Model caterpillars were made from soft modeling clay of eight colors (Figure 2) announced to be nontoxic and odorless (Chemical plant “Luch,” Yaroslavl, Russia), which had been provided to each researcher participating in the experiment. Colors were selected to cover the entire spectra from short‐wave to long‐wave, with black and white as not colored but contrasting in luminance. Model caterpillars of a standard size (25–30 mm length and 4–5 mm diameter) were built over a wire of 0.3–0.5 mm in diameter (Figure 2). Eight caterpillars (one of each color) were attached, individually, along thin branches of each of 15 selected trees (120 caterpillars at each site). The models were placed in the outer part of the crown at a height of 1.5–2 m and not less than 20 cm apart (Figure 3a). The two first inventories were conducted at three‐day intervals, whereas the following records were conducted at one‐ or two‐week intervals, depending on the intensity of predation. The only exception was the site in Georgia (Asia), where a single record was made 10 days after the establishment of the experiment. The total duration of the experiment was 64 days, on average, and varied from 10 to 118 days (Table A1) depending on the logistic circumstances of the observer. In ten of the 11 sites, the observations were long enough to account for seasonal changes in bird predation and preferences (described, e.g., by Mappes et al., 2014). During each record, all marks found on model caterpillars were attributed to a certain group of predators according to Low, Sam, McArthur, Posa, and Hochuli (2014), and marks of each type (Figure 3b–d) were counted. The models that had damage marks were remolded or replaced if the damage was severe.

### 2.2 Plasticine color analysis

A photograph of uniform clay pieces of all eight colors was taken in RAW format using a Canon 6D camera under daylight spectrum illumination with a white reflectance standard. The image was processed using Adobe Photoshop CC, and the mean camera‐specific RGB component values for each piece of plasticine were recorded.

**Figure 4:**
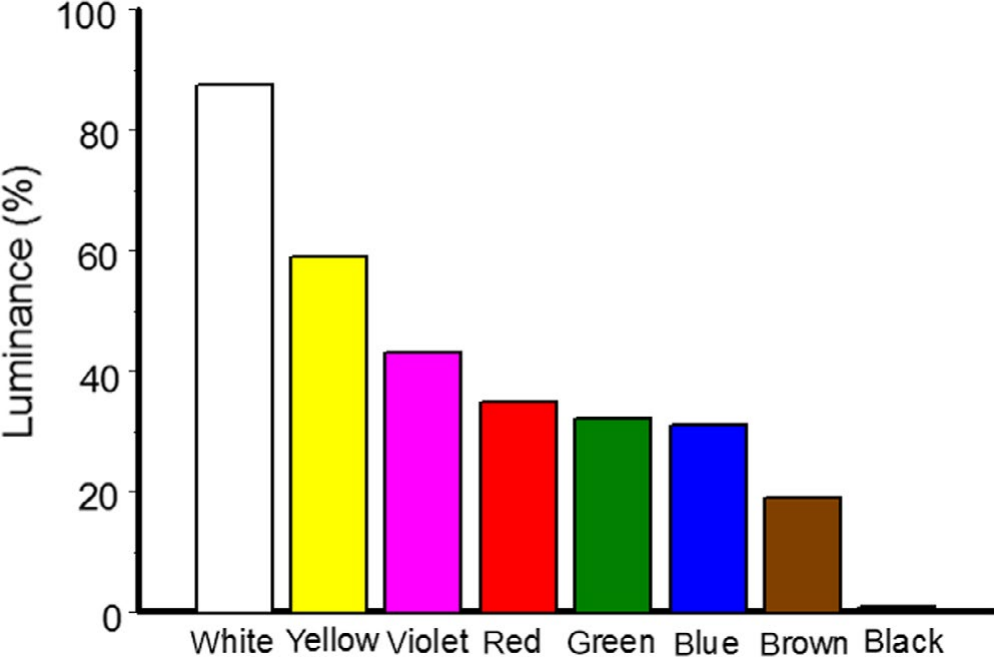
Relative luminance (percentage of the maximum component value, i.e., of 255) of eight colors of plasticine used in the experiment

To summarize the luminance independent (chromatic) color measures, the RG and BY ratios were calculated from camera‐specific RGB component values (Table A2 in Appendix), as follows: RG = (R − G)/(R + G); BY = (B − (R + G)/2)/(B + (R + G)/2) (Rothery, Scott, & Morrell, 2017). These ratios describe the redness versus greenness (RG) and blueness versus yellowness (BY) of each color. We also calculated the luminance (achromatic measure) of each color as (R + G + B)/3 (Rothery et al., 2017) and expressed it as a percentage of the maximum component value, i.e., of 255 (Figure 4). We expected multiple predator species to attack our models; consequently, we did not attempt to transform the RGB values into an avian or other animal color space.

### 2.3 Statistical analysis

Attack rates (separately by birds and by arthropod predators, and by all predators combined) were calculated as the sum of all attack marks by the respective category of predators on each model for the entire observation period, divided by the total length of the observation period in days. Attacks by mammal and reptile predators were too rare (see Section 3) to conduct separate analyses.

We analyzed the effects of different factors on predator attack rates by mixed model ANOVA (SAS GLIMMIX procedure; SAS, 2009) with climate zone (cold, temperate, and tropical), site nested within climate zone, color of model and color by climate zone interaction as fixed effects, and tree species nested within each site and tree individual nested within species as random effects. We increased the accuracy of the fixed effects *F* tests by adjusting the standard errors and denominator degrees of freedom using the latest version of the method of Kenward and Roger (2009). The significance of random effects was explored by a likelihood ratio test (Stroup, 2013). To evaluate latitudinal patterns, we calculated Pearson product–moment correlation coefficients between the estimated marginal means (obtained from mixed model ANOVA described above) of site‐specific attack rates on model prey of all colors together, as well as on models of two colors with most contrasting luminance (black and white).

The relative frequencies of predator attacks on models of different colors were quantified as the percentages of attacks on models of each color among the sum of attacks on models of all eight colors. The “preference” or “avoidance” was considered statistically significant if the recorded frequency of attacks differed (Fisher exact test, *p* < .05) from 0.125 (i.e., from the probability of attack expected at random). No arthropod attacks were recorded at three sites; therefore, the color‐specific attack rates for arthropod predators were estimated for eight sites only.

To account for possible directional changes in the intensity of predation in the course of the experiment due to birds learning that the artificial prey offer no nutritional reward (Mäntylä et al., 2008), we calculated site‐specific means of predation rates (attacks per day) for the first record (usually made after 3 days of exposure), for all other records, and for the last record separately, and compared these means by the signed‐rank test.

The associations between the frequencies of attacks on models of different colors with the chromatic (RG, BY) and achromatic (luminance) characteristics of those colors were explored by calculating Pearson product–moment correlation coefficients.

## 3 RESULTS

Of the 1,320 model prey exposed at the 11 sites, 531 (40.2%) were attacked at least once by birds, 366 (27.7%) by arthropods, 15 by mammals (1.1%), and 28 (2.1%) by other predators.

Across all study sites, attack rates of birds and arthropod predators based on the first record did not differ from either all other records combined (*S* = 0.5, *p* = 1 and *S* = 5.5, *p* = .57, respectively) or from the very last record (*S* = 1.5, *p* = .92 and *S* = 10, *p* = .11, respectively), indicating that predators did not learn to avoid plasticine prey.

**Figure 5:**
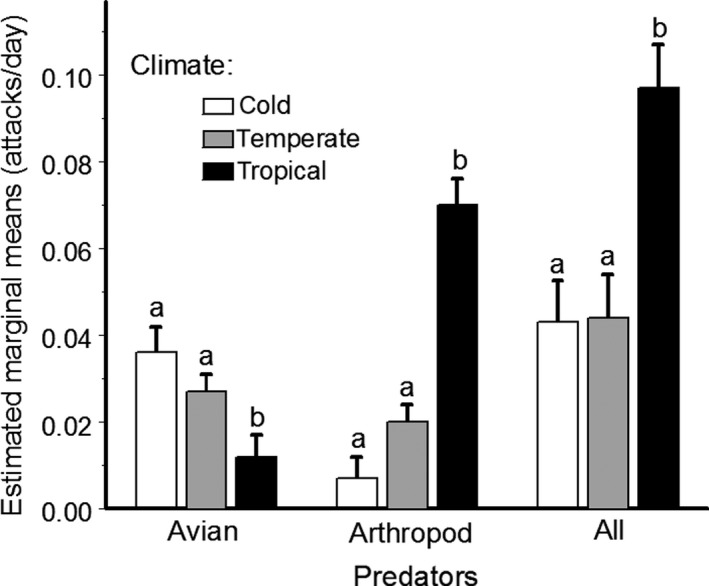
Attack rates (+ SE) of different groups of predators on plasticine models in cold, temperate, and tropical climates (all colors combined). Bars with different letters indicate significant (*p* < .05) differences between climates

The attack rates varied among the climate zones and among the sites nested within climate zones for both avian and arthropod predators, as well as for all predators combined (Table 1). Bird predation was lowest, whereas arthropod and total predation were highest in the tropics when compared with both cold and temperate sites (Figure 5). Model color influenced bird predation rates, but did not affect arthropod predation rates (Table 1). The attack rates on models of different colors varied among the climatic zones for bird predation, but did not vary for arthropod predation (interaction terms in Table 1). The attack rates by bird and arthropod predators, as well as by all predators combined, also varied among individual trees, while the effect of tree species was marginally significant for bird predation only (Table 1).

**Table 1:** Sources of variation in the attack rates on plasticine caterpillars of different colors (mixed model ANOVA, type III tests)

Effect	Source of variation	Birds		Arthropod predators		All predators	
		Test statistics	*p* value	Test statistics	*p* value	Test statistics	*p* value
Fixed	Climate zone	*F*_2, 22_ = 4.75	.019	*F*_2, 22_ = 35.8	<.0001	*F*_2, 22_ = 9.43	.0011
	Color	*F*_7, 1.134_ = 2.30	.025	*F*_7, 1.134_ = 1.89	.07	*F*_7, 1.134_ = 3.19	.0024
	Climate zone × Color	*F*_14, 1.134_ = 1.96	.018	*F*_14, 1.134_ = 0.85	.61	*F*_14, 1.134_ = 1.37	.16
	Site (Climate zone)	*F*_8, 22_ = 3.94	.005	*F*_8, 22_ = 2.76	.03	*F*_8, 22_ = 2.19	.07
Random	Tree species (Site)	*χ* ^2^_1_ = 3.40	.065	*χ* ^2^_1_ = 0.00	.98	*χ* ^2^_1_ = 1.91	.17
	Tree (Species × Site)	*χ* ^2^_1_ = 7.35	.0067	*χ* ^2^_1_ = 21.2	<.0001	*χ* ^2^_1_ = 34.5	<.0001

**Figure 6:**
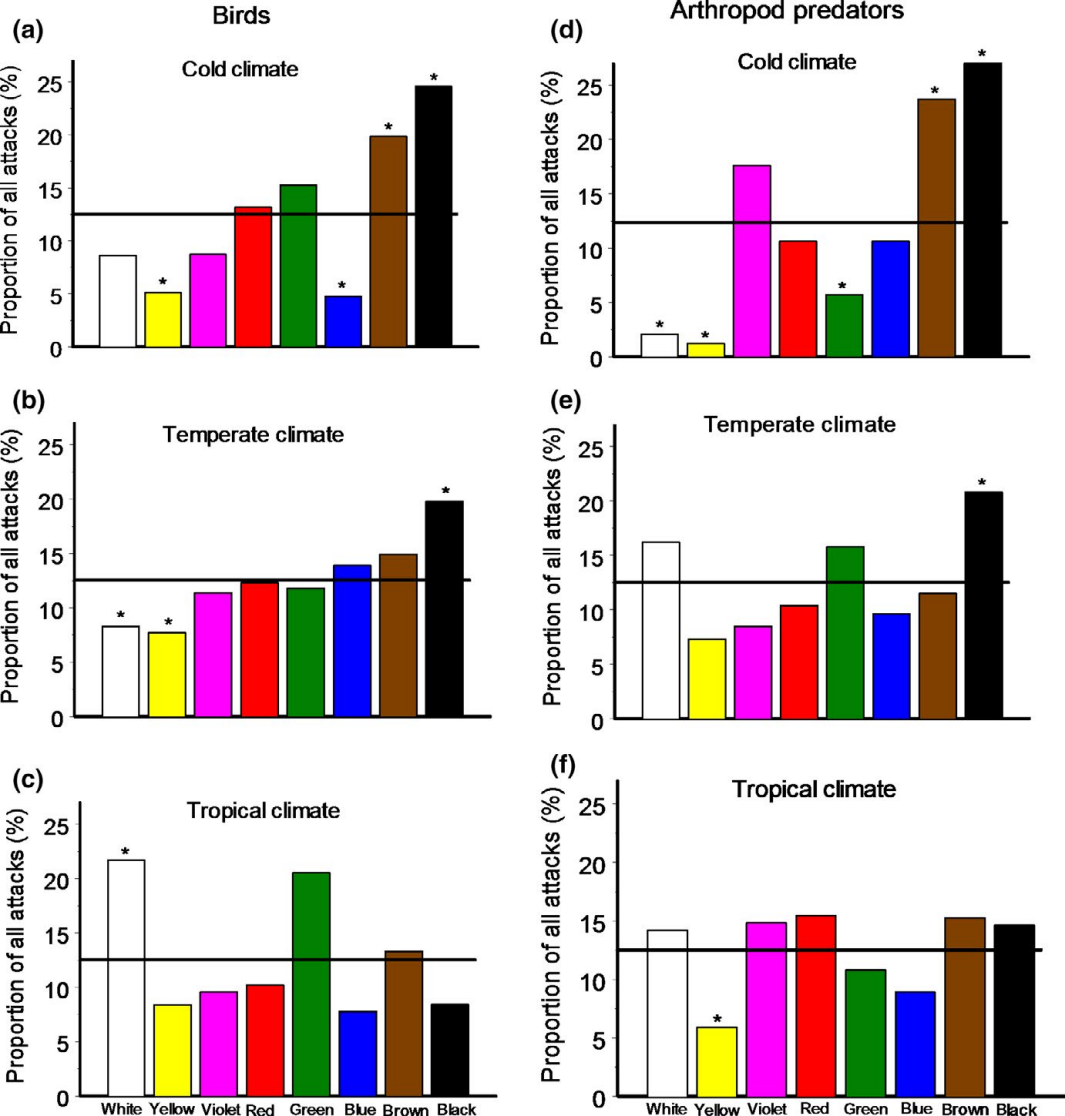
Distribution of predator attacks among different colors of model prey in different climates. Asterisks indicate significant (Fisher exact test, *p* < .05) differences from the equal probability distribution (shown by the horizontal line). Colors are ordered according to their luminance (consult Figure 4), from highest (white) to lowest (black)

When we compared the distributions of predator attacks among models of different colors, the proportions of predator attacks were highest on black and brown models in cold climates and on black models in temperate climates (Figure 6a,b,d,e). In tropical climates, birds disproportionally frequently attacked white models (Figure 6c), whereas arthropods attacked models of all colors (except for yellow) at similar rates (Figure 6f). Attacks on yellow models by both bird and arthropod predators were less frequent than would be expected at random in all climate zones (Figure 6a–f). The frequencies of predator attacks on models of other colors (green, blue, violet, and red) generally did not differ from those expected at random (Figure 6).

The proportions of attacks on models of different colors did not correlate with the chromatic characteristics of the models (BY and RG) neither for bird nor arthropod predators in any of the climate zones (data not shown). On the contrary, the probability of an attack on a model prey was negatively correlated with the achromatic characteristic (luminance) of the color in cold environments for both bird (*r* = −.78, *n* = 8 colors, *p* = .02) and arthropod predators (*r* = −.81, *n* = 8 colors, *p* = .02); in temperate environments for birds only (birds: *r* = −.93, *n* = 8 colors, *p* = .001; arthropods: *r* = −.26, *n* = 8 colors, *p* = .54), and was nonsignificant in tropical environments for either birds or arthropods (*r* = .24 and .24, *n* = 8 colors, *p* = .56 and .59, respectively).

**Figure 7:**
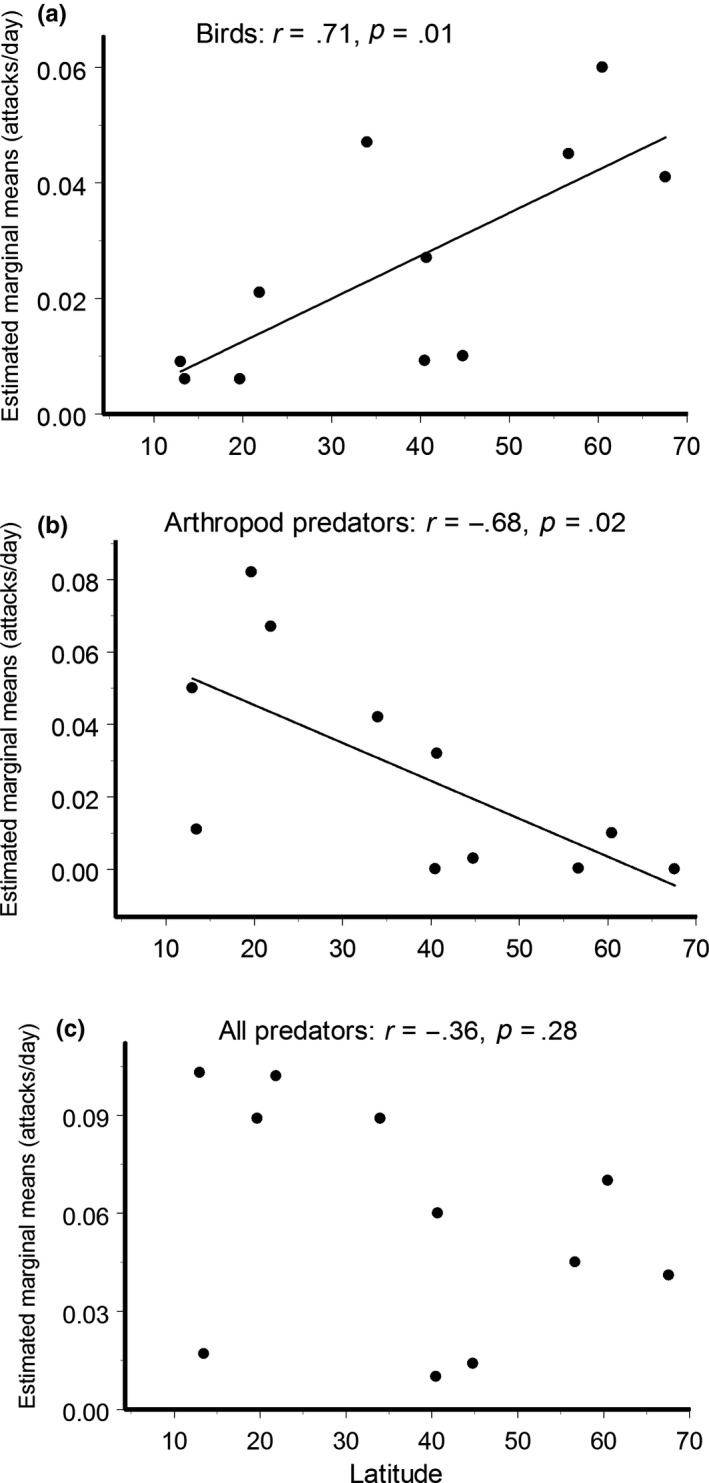
Correlation between predator attack rates (estimated marginal means; all colors summed for each tree) with the latitude of the site (a)—birds, (b)—arthropod predators, (c)—all predators (including birds, arthropods, mammals, and others)

The average site‐specific attack rates by birds (summed across model prey of all colors for the entire observation period) increased with latitude, while the arthropod predation decreased; as a result, when the attacks by all predators were combined, the correlation between predation and latitude appeared nonsignificant (Figure 7a–c). When these correlations were calculated based on the first record only (3 days in most sites), the correlation for bird predation became nonsignificant (*r* = −.25, *n* = 11 sites, *p* = .46), whereas the correlation for arthropod predators remained marginally significant (*r* = −.58, *n* = 11 sites, *p* = .06). The bird predation rates on black and on white model prey (extremes in luminance) demonstrated different latitudinal patterns: The attack rates on black models significantly increased with an increase in latitude (*r* = .69, *n* = 11 sites, *p* = .02), whereas the attack rates on white models did not correlate with latitude (*r* = .08, *n* = 11 sites, *p* = .81).

## 4 DISCUSSION

### 4.1 Effects of model color on predator attacks

The increasing use of artificial caterpillars in ecological studies, and particularly in those studies addressing spatial patterns in predation rates (reviewed by Lövei & Ferrante, 2017), underlines the urgent need to learn how the characteristics of a model prey influence its attractiveness for predators in different environments. The exposure of differently colored model prey in multiple sites around the globe has allowed us to uncover interactive effects of environmental properties and prey color on predator attack rates.

Our finding of differential responses of birds to colors of model prey is in agreement with our expectations, because birds possess high capabilities for color discrimination, and the decisions regarding the suitability of food items made by both frugivorous and insectivorous birds greatly depend on food coloration (Théry & Gomez, 2010). However, negative correlation between probability of bird attack and prey luminance in cold and temperate climates, but not in tropical climate and, consequently, the different latitudinal trends in the frequencies of bird attacks on prey with contrasting luminance, to our knowledge, have not been reported earlier. We suggest that these discovered differential responses of birds to model luminance are associated with differences in the light regimes between the high‐ and low‐latitude environments: In tropical forests, only 1%–5% of light transmits through the canopies, while in boreal forests it may be as high as 65% (Messier, Posada, Aubin, & Beaudet, 2009). In sparse boreal and temperate forests, a high brightness contrast between black prey and the visual background increases the probability of detection and elicits attacks by predators (Théry & Gomez, 2010). By contrast, in the shady understorey of dense tropical forests, white models showing the highest luminance were the most frequently attacked by birds. This finding is in line with the results of Cheng et al. (2018), who found that black butterfly models in tropical forests experienced lower predation rates in shady habitats than in open habitats, whereas white models showed the opposite pattern.

In our tropical sites, models of different colors were generally attacked at similar rates (except for the brightest, most attacked, white models), which may be explained by a decreased ability of birds to discriminate colors in the understory of tropical forest due to low light intensity (Gomez et al., 2014; Olsson, Lind, & Kelber, 2015). Similar attack frequencies on prey of different colors in our tropical sites might also be attributable, in part, to a higher variability in coloration of insects in the tropics, as demonstrated, for example, for butterflies (Adams et al., 2014). Birds in tropical forests are therefore faced with a higher variety of prey colors than birds in temperate and boreal forests, where models of some colors can be rejected by birds due to neophobia, that is, the avoidance of an object solely because it has never been experienced (Greenberg & Mettke‐Hofman, 2001). Birds growing in diverse environments are known to exhibit decreased neophobia (Greenberg & Mettke‐Hofman, 2001); therefore, birds living in tropical forests with extremely high biodiversity (Willig et al., 2003) may accept prey of a wider range of colors and patterns.

Yellow models were consistently attacked at low rates by birds across all our sites. Yellow coloration, along with other long‐wavelength colors, is an effective warning signal, in particular because yellow is highly conspicuous when viewed against green foliage across a variety of habitats (Stevens & Ruxton, 2012). Birds are usually not attracted by yellow fruits (Sinnott‐Armstrong et al., 2018) and avoid yellow bird feeders (Rothery et al., 2017). Interestingly, we found that yellow models were also attacked at low rates by arthropod predators in all climatic zones, indicating that yellow coloration provides effective and universal protection for prey against diverse predators in forests across a large latitudinal gradient.

Our results suggest that the strongest difference in the probability of bird attack on model prey was observed for colors that most contrasted in luminance, whereas the probability of attacks on other colors did not differ from random expectation. Taking these results together with the observed lack of correlations between prey attack rates and the chromatic characteristics of models indicates that the luminance of the prey, rather than the color itself, is the most important determinant of predation rates in environments varying in background characteristics and ambient light availability. Similarly, Cheng et al. (2018) concluded that tropical habitats that differ in light regime can have contrasting effects on prey luminance and therefore on predation risk. Luminance is especially important in the spatial vision of birds (Stevens & Cuthill, 2006), and birds generally avoid objects with high reflectance. For example, tits show an initial avoidance of glossy prey (Doktorovová et al., 2019; Waldron et al., 2017). The brightness contrast between prey and background, rather than the color contrast, may function as a warning signal also for color‐blind predators (Prudic, Skemp, & Papaj, 2007).

Many invertebrate predators possess color vision (Briscoe & Chittka, 2001), and some even use prey colors in their foraging tactics (Taylor et al., 2014). However, among other cues, colors generally do not play an important role in prey detection and discrimination by arthropod predators (Zvereva & Kozlov, 2016). Ants, for example, which contribute to the vast majority of arthropod predation in the tropics (Sam, Remmel, & Molleman, 2015), primarily use chemical cues in their foraging behavior (Traniello, 1989). This explains the nonsignificant effects of prey model color on the attack rates of arthropod predators across climates. Nevertheless, in cold climates, low‐luminance models (black and brown) had a higher probability of arthropod predation, while high‐luminance models (white and yellow) had a lower probability (Figure 4d). This indicates that the nonchromatic characteristics of coloration affect the detectability of prey not only by birds, but also by arthropod predators in high light environments, where low‐luminance colors are most conspicuous. Thus, prey luminance may constitute an important factor affecting overall prey mortality.

The greater number of marks left by arthropod predators on dark models in cold climates may also reflect, in part, the effects of temperature on modeling clay. The plasticine becomes harder at low temperatures, so the visibility of arthropod predation marks decreases with decreases in ambient temperature (Muchula, Xie, & Gurr, 2019). Darker objects also heat up more rapidly in sunlight when compared with paler objects, and the difference in temperatures between black and light‐colored objects increases with decreasing air temperature (Clusella Trullas, Wyk, & Spotila, 2007). Therefore, the marks of arthropod predators in cold climates may be stronger, and therefore easier to distinguish, on black and brown models than on models of colors with higher luminance.

We conclude that attack rates on model prey of different colors varied geographically and according to predator identity (Table 1, Figure 4). This supports the notion that properties of the visual background and ambient light conditions, together with differences in species composition, perceptive abilities, experiences, preferences, and demands of predators, as well as in the diversity of potential prey, can modify selection and the relative protective values of prey color patterns (Endler, 1993; Greenberg & Mettke‐Hofman, 2001; Ruxton et al., 2018; Prudic et al., 2007; Wennersten & Forsman, 2009; Théry & Gomez, 2010). It remains to be investigated whether distributions of different colors across natural communities of insect larvae parallel the spatial differences in selection indicated by our results, as previously demonstrated in other systems (Karpestam, Merilaita, & Forsman, 2013).

### 4.2 Variation in the intensity of predator attacks among tree species and among individual trees

We found a marginally significant variation in bird predation rates among tree species on which our models were attached. This result is in line with the studies of Muiruri, Rainio, and Koricheva (2016) and of Wennersten and Forsman (2009) and may be explained by the strong foraging preferences of insectivorous birds for certain tree species (Gabbe, Robinson, & Brawn, 2002; Holmes & Robinson, 1981). In addition, variation in canopy structure among tree species, including differences in crown density, complexity, and color, may affect both the detectability and the prey accessibility (Muiruri et al., 2016; Šipoš & Kindlmann, 2013). Furthermore, both bird and arthropod predation significantly varied among individual trees, presumably due to both the obvious environmental heterogeneity of each study site and the position of a particular tree in relation to the foraging territories of predators (e.g., its nearness to bird and ant nests).

Thus, natural variation calls for the use of several plant species and considerable numbers of microhabitat replicates when conducting macroecological studies employing artificial prey.

### 4.3 Geographic variation in the intensity of predator attacks

In line with the study by Roslin et al. (2017), we found that attack rates by arthropod predators on model prey decreased from low to high latitudes. The tropics are habitats with a very high abundance and diversity of arthropod predators, especially ants and wasps, which dominate the predator communities in tropical forest understoreys (Floren, Biun, & Linsenmair, 2002; Sam et al., 2015). More than a half of the studied ant species attack large caterpillars (Floren et al., 2002), and experiments with live insect prey have demonstrated higher predation rates by ants in the tropics than in temperate forests (Jeanne, 1979). Thus, the pattern revealed using plasticine caterpillars is likely to be a reliable reflection of the real latitudinal trends in arthropod predation on insect prey, which is shaped mostly by high arthropod predation in tropics.

However, a recent study showed a decreased visibility of ant attack marks when the attack took place at low temperatures (below 8°C), whereas no effect of temperature was observed on the probability of identifying visible attack marks when the attacks took place between 16 and 32°C (Muchula et al., 2019). This raised the possibility that part of arthropod predator attacks in cold climates, where temperatures during the summer season are frequently below 16°C, did not leave any visible marks on the plasticine models, thereby leading to an underestimation of arthropod predation rates at high latitudes. This potential bias may have partly contributed to the geographical pattern in arthropod predation observed in our study (Figure 6b) and in the study by Roslin et al. (2017), making the detected poleward decline steeper than it might be when investigated using natural prey.

The direction of the latitudinal gradient in bird predation rates, which we found to be lowest in tropical sites, was in strikingly contrast to our expectations. The considerably higher density of insectivorous birds and the greater biomass of arthropods consumed by birds per hectare in tropical forests relative to temperate and boreal forests (Nyffeler et al., 2018) points to the strongest bird predation pressure on herbivorous insects in tropical forests. Nevertheless, the predation rates measured using model prey demonstrate either an absence of any latitudinal trend (Roslin et al., 2017) or a significant poleward increase in bird predation (this study).

This discrepancy may be explained by several factors. First, most of the studies on spatial patterns in predation conducted to date, including our study, employed prey items placed within reach of the observer (Lövei & Ferrante, 2017), for example, below 1 m in the study by Roslin et al. (2017). This may lead to an underestimation of bird predation in tropical forests, where both bird enclosures (van Bael, Brawn, & Robinson, 2003) and model caterpillars (Loiselle & Farji‐Brener, 2002) revealed higher rates of bird predation in the top canopy than in the lower forest strata. Model caterpillars placed in tropical forest understoreys sometimes showed no signs of bird predation (Sam et al., 2015). These results may be at least partly due to the higher abundance of natural prey in the top canopy than in the understorey of a tropical forest (van Bael et al., 2003; Basset et al., 2015).

Second, low bird attack rates in tropical forest understoreys may be explained by the low light intensity under the canopies of dense tropical forests, which may impair prey detectability (Gomez et al., 2014; Olsson et al., 2015). Thus, bird predation measured in the understoreys of tropical forests may be biased toward underestimation of habitat‐specific values, and this bias could contribute to the latitudinal pattern observed in our study. Although experimenting in top canopies is logistically challenging, we call for more studies comparing predation pressure on herbivorous insects in top canopies and understoreys in different geographic zones to obtain a more accurate estimate of habitat‐specific predation values. The understanding of changes in biotic interactions across the vertical dimension is important in explaining global biodiversity patterns, particularly those associated with environmental gradients, including disturbance, latitude, and elevation (Nakamura et al., 2017).

Despite the many potential confounding factors, a poleward increase in bird attacks on plasticine models may still reflect a real latitudinal pattern of bird predation on herbivorous insects. The densities of insectivorous birds may decrease with latitude at a lower rate than the density of potential prey. Also, a high abundance of alternative food in the tropics, such as fruits and nonherbivorous arthropods (e.g., ants and spiders; Cardoso, Pekár, Jocqué, & Coddington, 2011; Floren et al., 2002), may decrease the predation pressure upon herbivorous insects.

One possible reason why we detected a significant poleward increase in bird predation, while Roslin et al. (2017) did not find any latitudinal pattern, is that the prey exposure duration was much longer in our study than in the study by Roslin et al. (2017) (64 vs. 4 days, on average, respectively). This possibility is supported by lack of a correlation between latitude and bird predation estimates based on the first record only, when our models were exposed for only 5.4 days, on average. Considerable seasonal variations in predation rates related to the breeding season of the most abundant insectivorous birds (e.g., Mappes et al., 2014; Remmel & Tammaru, 2009) may distort geographical patterns when the start of an experiment in habitats that differ in seasonality is not adjusted precisely to a certain stage of the breeding season. Our study, in line with Muiruri et al. (2016), showed that the rates of bird attacks on plasticine models did not decrease with the time of exposure, probably because avoidance learning of prey usually develops when associated with irritating or toxic compounds, while taste of plasticine is presumably neutral. Therefore, we conclude that exposure of prey during several weeks or even several months is critical for revealing macroecological patterns in bird predation on herbivorous insects, while for arthropod predation, the duration of the experiment appeared less important than it was for bird predation.

The paramount methodological advantage of our study that allowed the detection latitudinal patterns in bird predation was the use of model prey of different colors. Our results suggest that attack rates on the prey are differently affected by prey color in different environments and that summation of attacks on prey of different colors mitigates the effects of the environment on the probability of attack on a certain prey type. Thus, we are of the opinion that our experimental design provided more realistic site‐specific estimates of bird predation rates when compared to the use of prey of one color across different environments. We conclude that bird predation on herbivorous insects increases from the equator toward the poles, in an opposite direction to arthropod predation and opposite the predictions of the Latitudinal Biotic Interaction Hypothesis (Schemske et al., 2009). The contrasting latitudinal patterns found in the attack rates of bird and arthropod predators, thereby weaken the overall latitudinal trend in top‐down control of herbivorous insects.

## CONFLICT OF INTEREST

None declared.

## AUTHOR CONTRIBUTIONS

ELZ and MVK planned the study and wrote the first draft of the manuscript. ELZ, BC, TC, AF, JAH, LP, VP, NS, KJT, GX, VZ, and MVK performed the experiments. TK and MVK conducted statistical analyses. ELZ led the writing of the manuscript, and all authors contributed critically to the drafts and gave final approval for publication.

## Data Availability

Data available from the Dryad Digital Repository: https://doi.org/10.5061/dryad.tx95x69sx

## APPENDIX 1

**Table A1:** Basic information on field experiments performed worldwide

Site, country	Coordinates and altitude	Vegetation type	Mid‐summer temperature, °C	Climate	Plant species	Start date	Finish date	Number of censuses	Responsible researcher
Sacsayhuaman, Peru	13°30′20″S, 71°58′37″W 3,550 m a.s.l.	Andean tropical forest	12.9	Cold	*Escallonia resinosa, Polylepis incana, Polylepis racemosa*	17‐Jul‐2018	19‐Oct‐2018	14	N. Salinas
Apatity, Russia	67°38′21″N, 32°45′04″E 150 m a.s.l.	Subarctic taiga forest	14.3	Cold	*Betula pubescens, Pinus sylvestris, Salix myrsinifolia*	11‐Jun‐2018	25‐Aug‐2018	3	V. Zverev, M. Kozlov
Kustavi, Finland	60°31′58″N, 21°18′08″E 10 m a.s.l.	Taiga forest	16.5	Cold	*Sorbus aucuparia, Betula pubescens, Pinus sylvestris*	28‐May‐2018	17‐Sep‐2018	9	E. Zvereva
Kalmar, Sweden	56^o^42′39″N, 16^o^22′18″E, 3 m a.s.l.	Mixed temperate forest	17.5	Temperate	*Sorbus aucuparia, Quercus robur, Betula pubescens*	24‐May‐2018	20‐Jul‐2018	8	A. Forsman
Cestas, France	44°45′14″N, 0°42′36″W, 50 m a.s.l.	Mixed broadleaved temperate forest	20.2	Temperate	*Betula pendula, Quercus robur, Castanea sativa*	23‐May‐2017	1‐Sep‐2017	10	B. Castagneyrol
Mtirala, Georgia	40°40′37″N, 41°52′23″E 300 m a.s.l.	Mountain broadleaved forest	20.7	Temperate	*Alnus glutinosa* var. *barbata*, *Castanea sativa, Carpinus orientalis*	19‐Sep‐2018	29‐Sep‐2018	1	V. Zverev, M. Kozlov
Stellenbosch, South Africa	33°57′27″S, 18°55′16″E 270 m a.s.l.	Renosterveld‐fynbos ecotone	21.2	Temperate	*Pterocelastrus tricuspidatus, Dodonaea viscosa angustifolia, Protea nitida*	1‐Nov‐2018	2‐Dec‐2018	5	K. J. Theron
Ermita de Navahonda, Spain	40°26′40″N, 4°14′46″W 842 m.a.s.l.	Mediterranean woodlands	24.2	Temperate	*Quercus ilex, Cistus ladanifer, Acer monspessulanum*	5‐Mar‐2018	3‐Jul‐2018	17	V. Polo, J. A. Hernández ‐Agüero
Fazenda Tanguro, Mato Grosso, Brazil	13°04′27″S, 52°22′40″W 385 m a.s.l.	Tropical evergreen forest	25.7	Tropical	*Mabea fistulifera, Sclerolobium paniculatum, Myrcia multiflora*	3‐May‐2018	11‐Jun‐2018	6	L. Paolucci
Xishuangbanna, China	21°55′05″N, 101°16′26″E 570 m a.s.l.	Tropical rainforest	26.2	Tropical	*Meiogyne* sp., *Leea compactiflora*, *Drypetes salicifolia*	6‐Nov‐2018	16‐Dec‐2018	3	G. Xu
Parque Estadual do Rio Doce, Brazil	19°42′23″S, 42°34′33″W 270 m a.s.l.	Atlantic forest	26.6	Tropical	*Adenocalymma subsessifolium, Sorocea guilleminiana, Casearia selloana*	28‐Sep‐2017	4‐Nov‐2017	5	T. Cornelissen

**Table A2:** Color characteristics of plasticine used in the experiment (estimated by Adobe Photoshop CC from photographs made with Canon 6D camera)

Plasticine color	Component values		
	Red	Green	Blue
Black	6	6	15
Blue	0	100	136
Brown	124	24	0
Green	0	162	84
Red	206	23	39
Violet	136	49	143
White	219	214	222
Yellow	212	194	48
